# Supplementary material for: DeCAF: a novel method to identify cell-type specific regulatory variants and their role in cancer risk
Source: Genome Biol. 2022 Jul 8;23:152. doi: 10.1186/s13059-022-02708-9 (PMC9264694; doi:10.1186/s13059-022-02708-9)
Supplement: Supplementary file 2 — Additional file 2 Supplementary Figures. [file 13059_2022_2708_MOESM2_ESM.pdf]

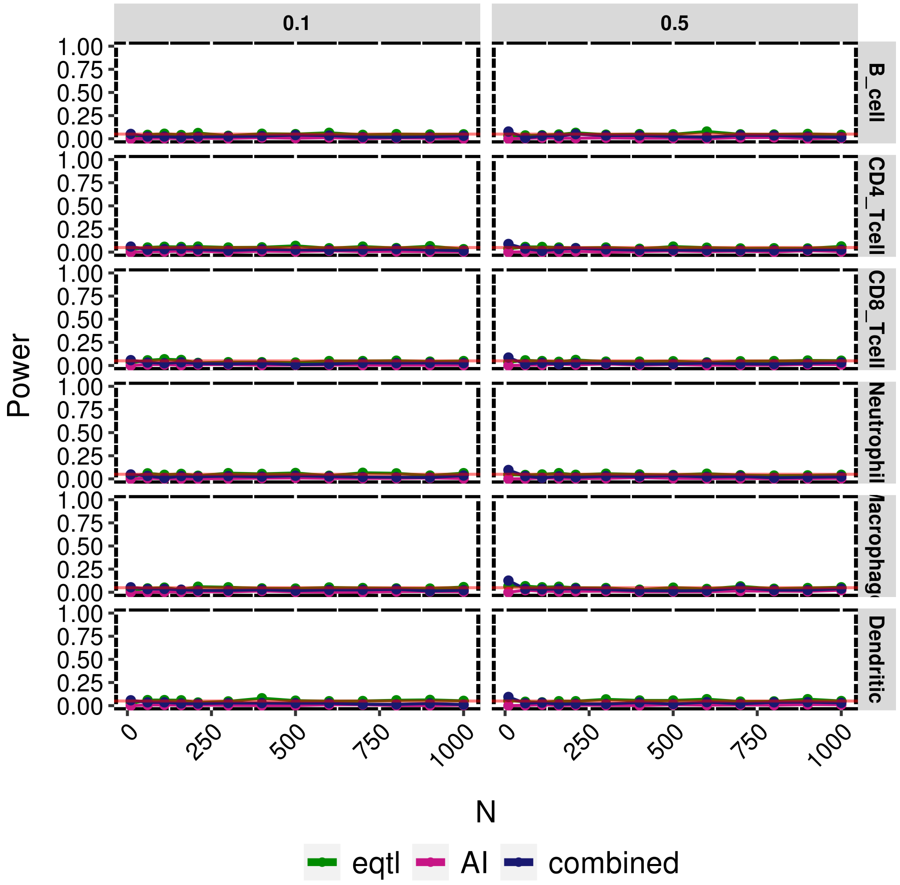

Figure S1 cfQTL models for simulated data from a uniform distribution. A) Plot depicting the power (y-axis) for varying number of individuals (x-axis) and either eQTL (green), AI (pink), or DeCAF combined method (blue) at a null allelic fraction (effect size of AI) and MAF (columns). B) Plot depicting the power (y-axis) for varying number of individuals (x-axis) and either eQTL (green), AI (pink), or DeCAF combined method (blue) at different allelic fractions (rows; effect size of AI) and MAF (columns).

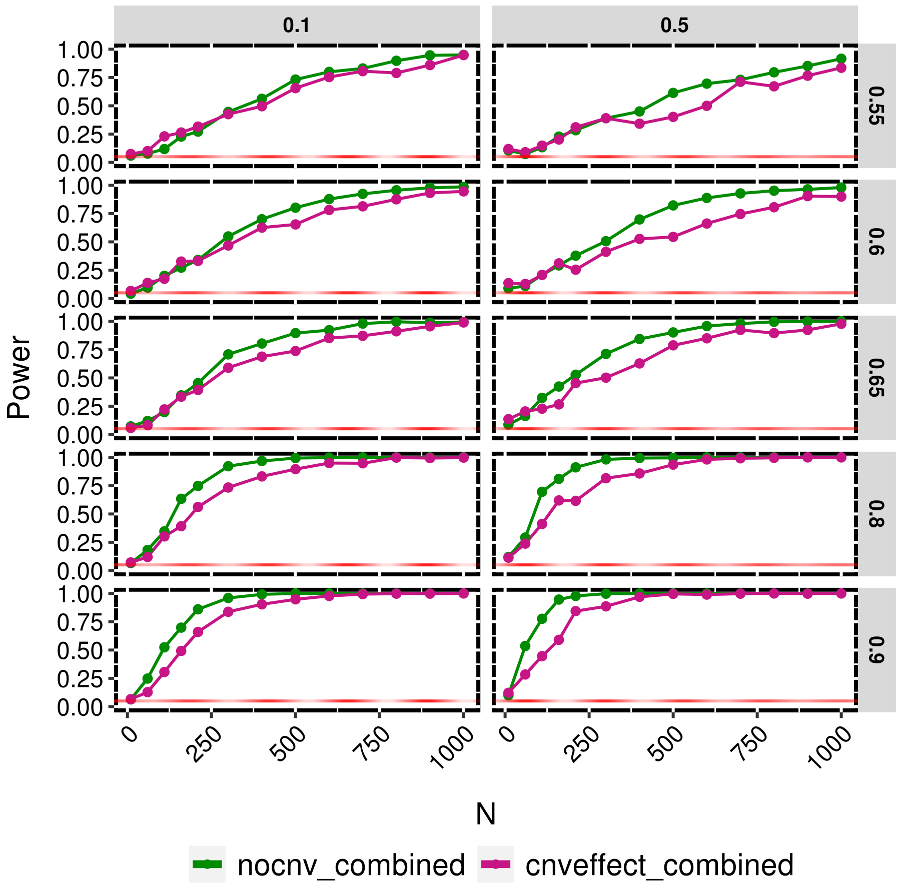

Figure S2 Null cfQTL models for simulated data using TIMER cell fractions. Plot depicting the power (y-axis) for varying number of individuals (x-axis) and either eQTL (green), AI (pink), or DeCAF combined method (blue) at a null allelic fraction (effect size of AI) and MAF (columns).

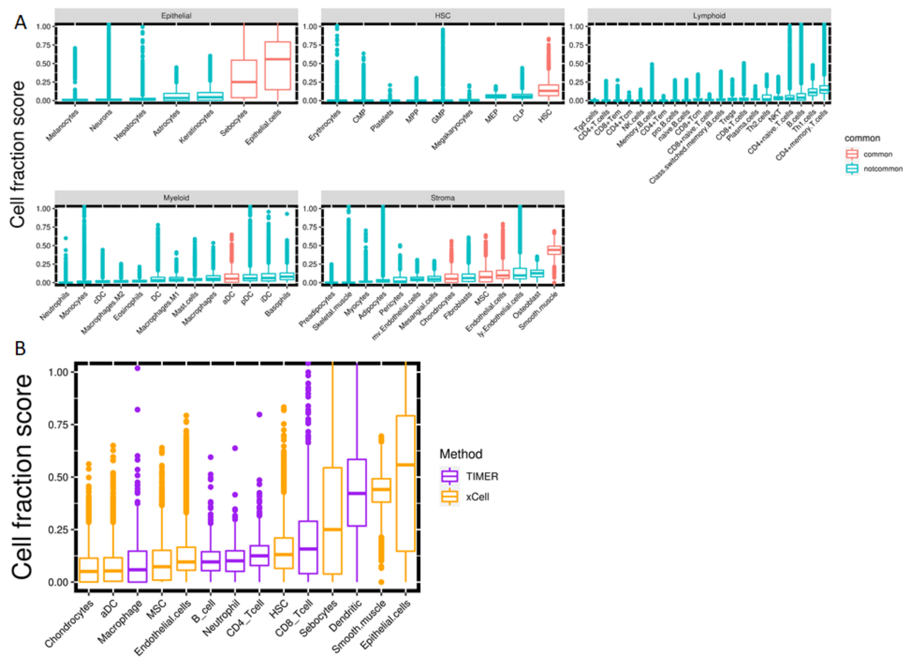

Figure S3 Comparing cfQTL models with or without CNV for simulated data. Plot depicting the power (y-axis) for a varying number of individuals (x-axis) and either no CNV in the DeCAF testing model (green) or CNV in the DeCAF testing model (pink). Each column represents a different MAF and each row is a different allelic fraction (effect size of AI).

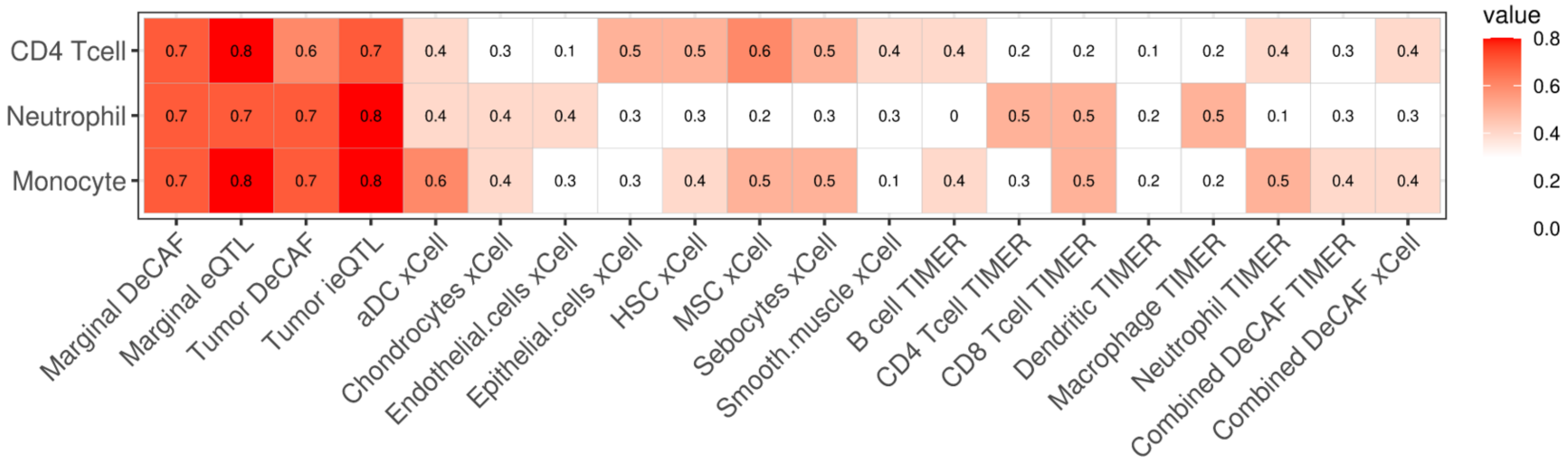

Figure S4 Deconvoluted cell fractions. A) Plot depicting the range of calculated cell fractions from xCell and TIMER deconvolution methods for TCGA KIRC tumors. B) All cell type fractions are shown for xCell and a cell fraction score inter quartile range (IQR) > 0.1 are in pink.

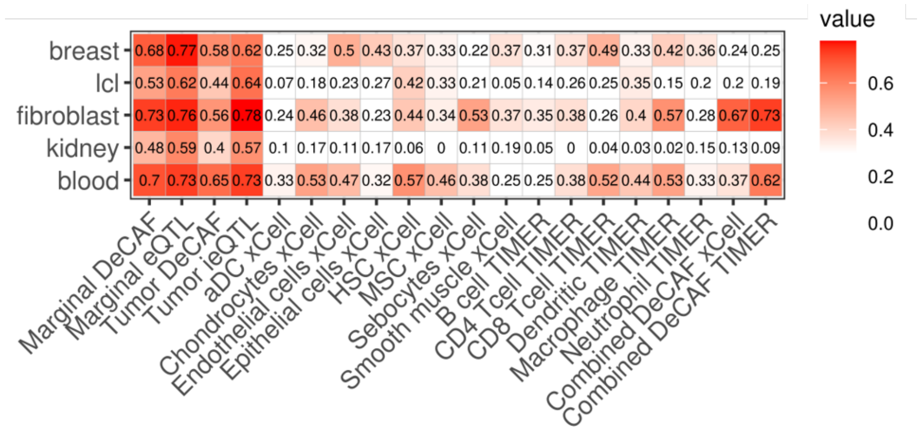

Figure S5 Removal of mapping bias. A) Volcano plot of allelic fraction (x axis) vs marginal DeCAF p-value (y axis, log10 scale). B) Violin plot of allelic fraction (y axis) split by if the variant was marginal DeCAF significant (FDR 10%, x axis).

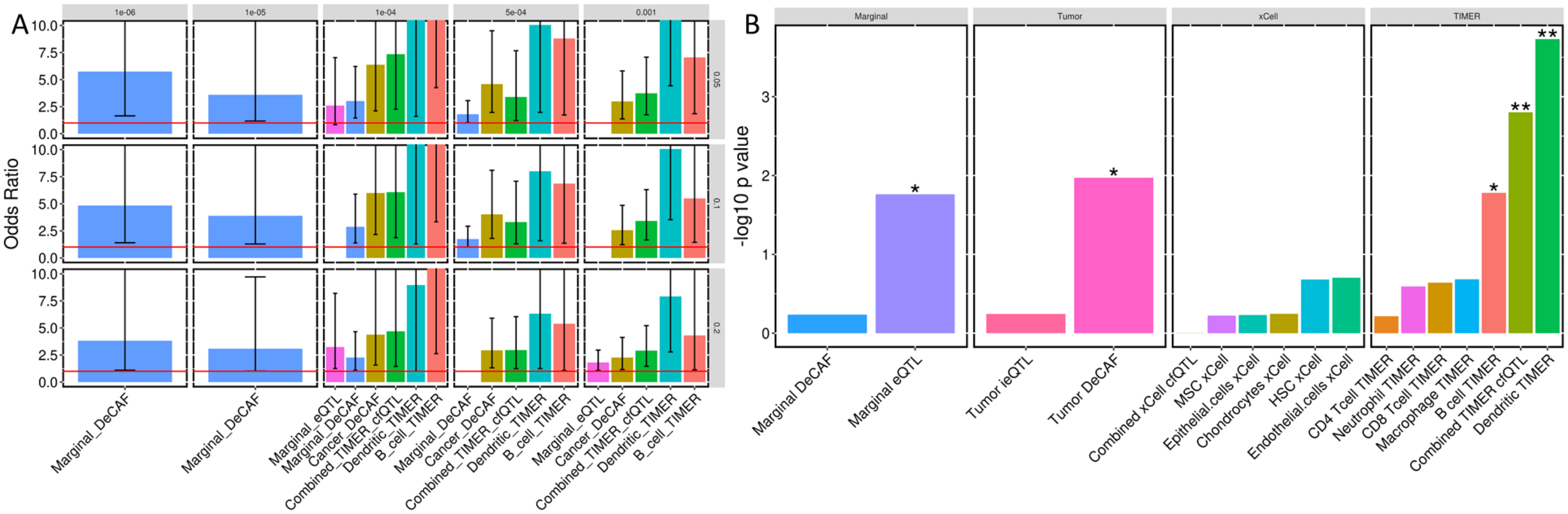

Figure S6 Comparing eQTL and AI effect sizes. Plot depicting the eQTL (y-axis) for a AI (x-axis) and significance of the QTL effect in marginal (FDR 10%) for both eQTL and AI (green), AI only (pink), or eQTL only (blue). Spearman ρ correlation and p-value in corner of plot.

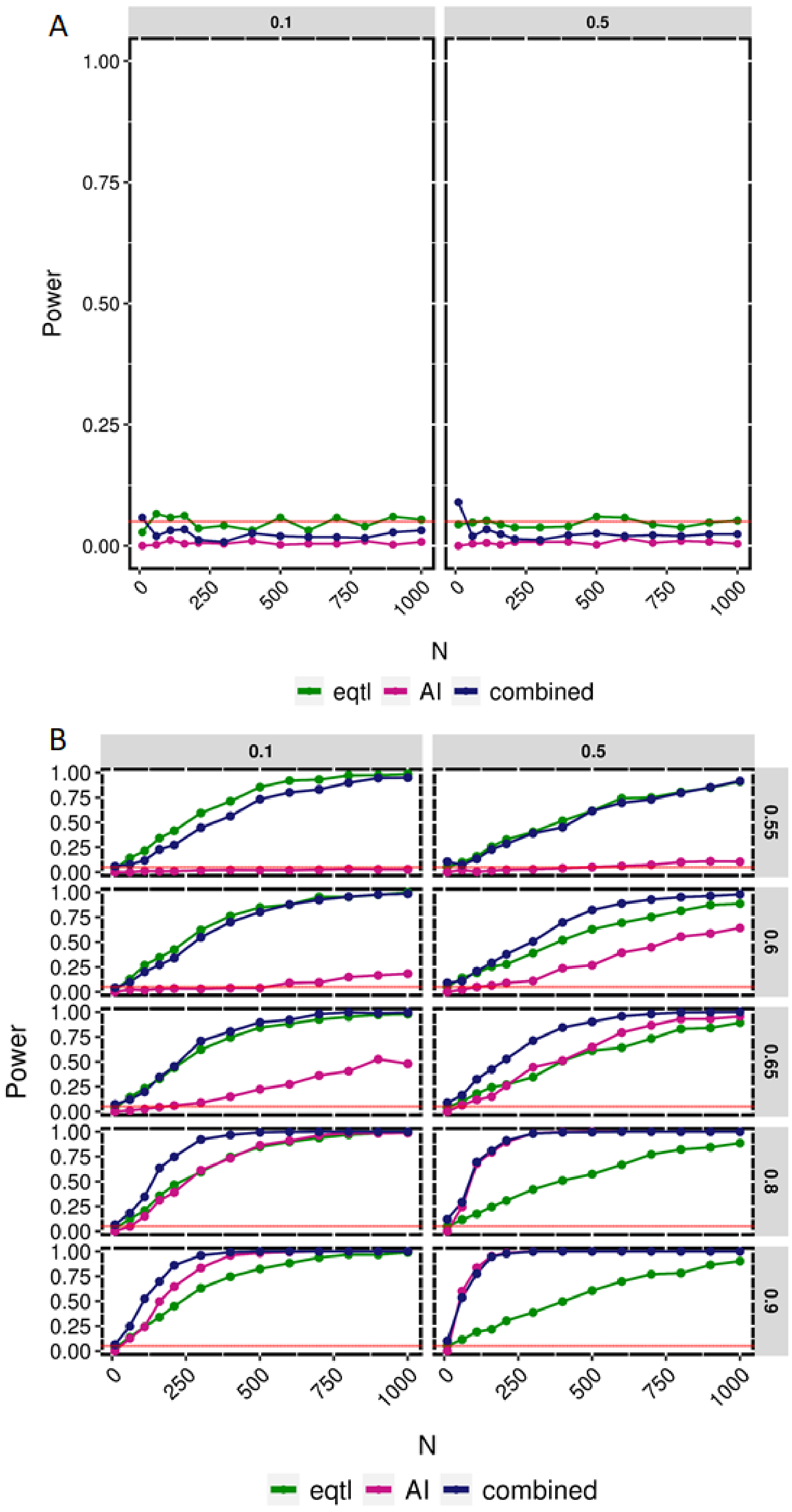

Figure S7 TCGA KIRC normal tissue cfQTL eGenes. Comparing eGenes from DeCAF vs standard interaction QTL (ieQTL) methods. Plot depicting the number of significant cfQTL genes (y-axis) for each method (x-axis).

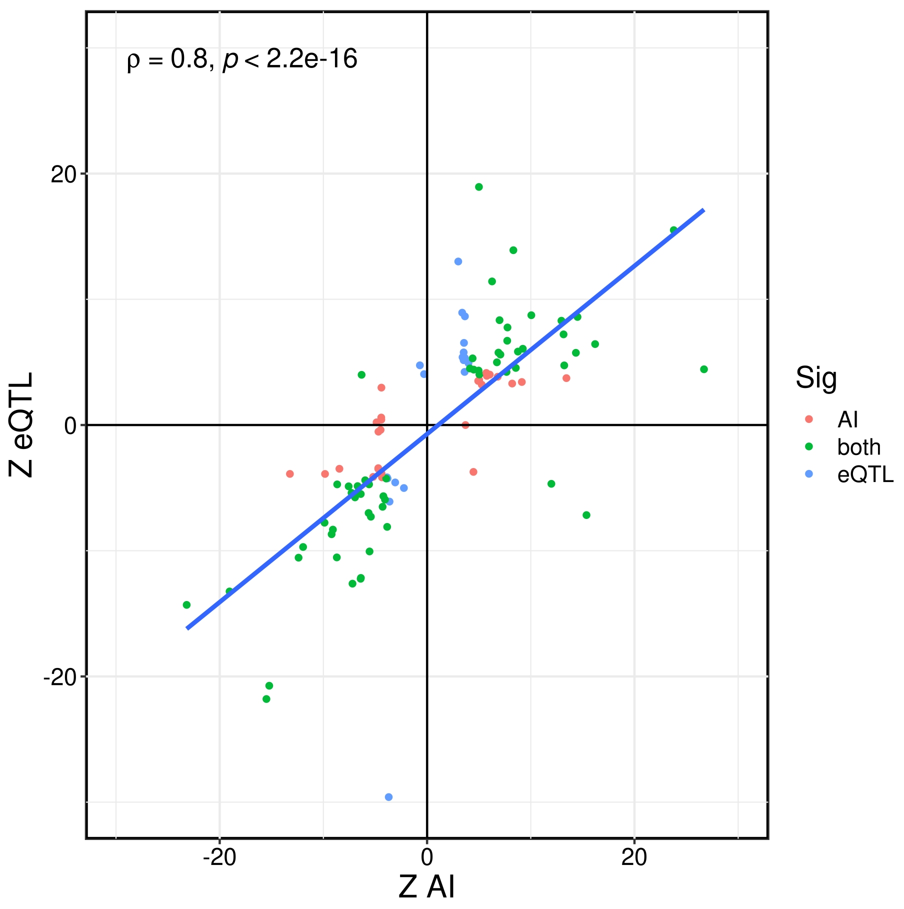

Figure S8 Comparing FDR thresholds for number of significant cfQTLs identified. Plot depicting the number of significant cfQTL genes (y-axis) for each cell type (x-axis) for increasing FDR thresholds.

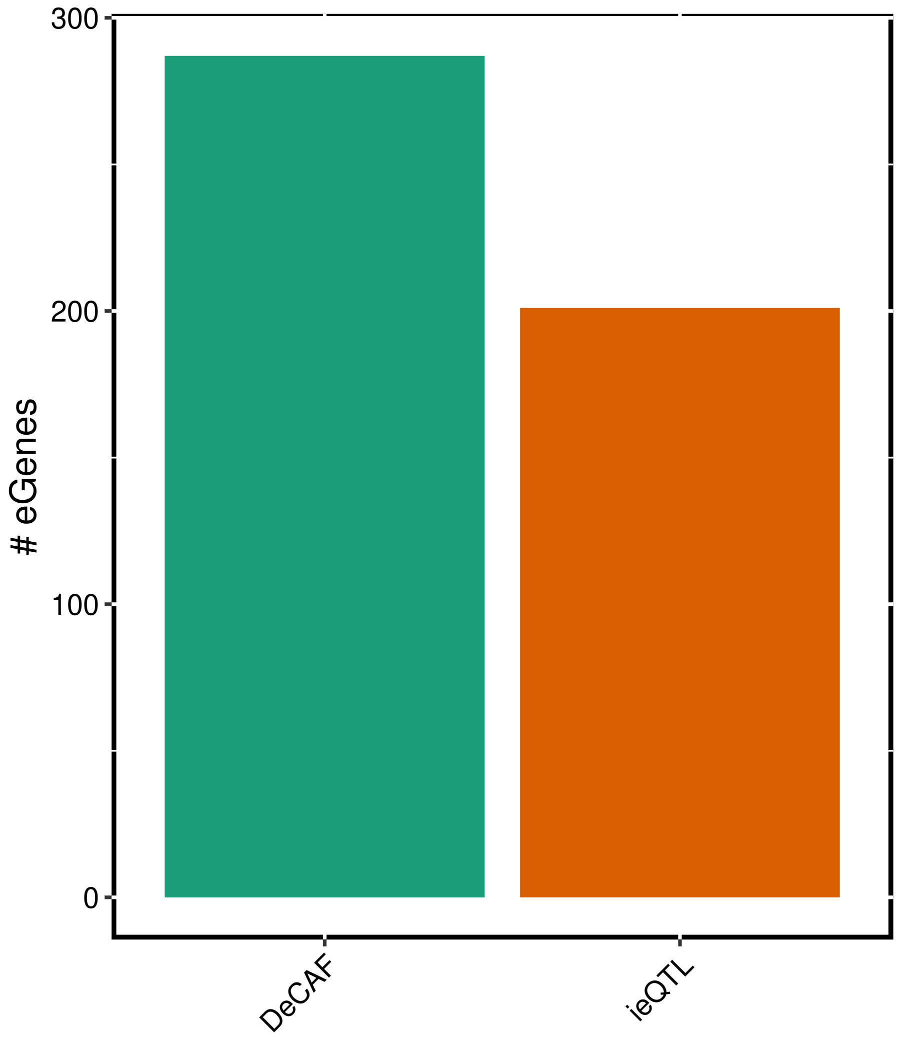

Figure S9 BLUEPRINT Replication. A) Average Z^2 replication of marginal, cancer, and TIMER and xCell cfQTLs in BLUEPRINT. Insignificant results (crossed out values) are based on p-value>0.05. B) π_1 replication of marginal, cancer, and TIMER and xCell cfQTLs in BLUEPRINT.

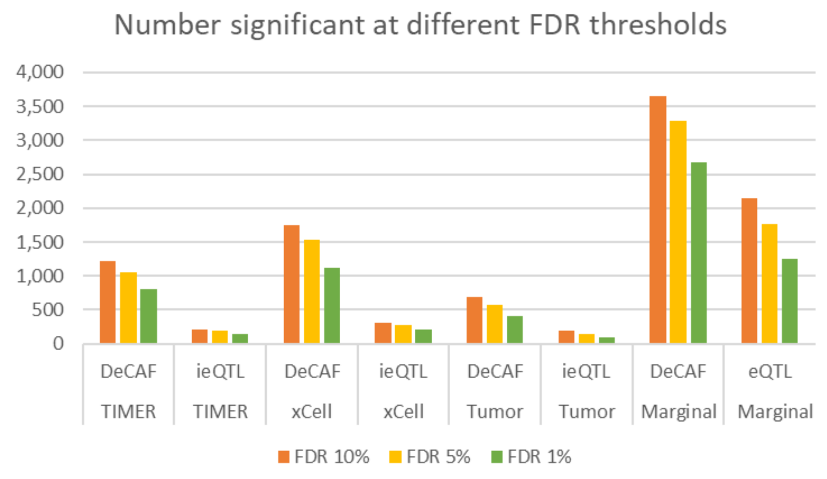

Figure S10 GTEx Replication. A) Average Z^2 replication of marginal, cancer, and TIMER and xCell cfQTLs in GTEx. Insignificant results (crossed out values) are based on p-value¿0.05. B) π_1 replication of marginal, cancer, and TIMER and xCell cfQTLs in GTEx.

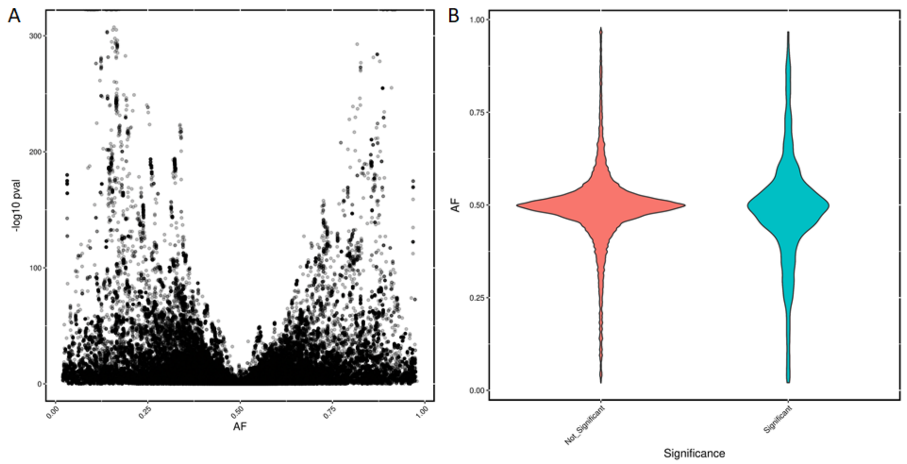

Figure S11 Enrichment of cfQTLs in GWAS. A) Significant enrichment of cfQTLs (FDR thresholds rows) in RCC GWAS (p-value thresholds columns) from a fisher's test. B) Significant enrichment of cfQTLs (FDR 20%) in RCC GWAS (p-value$<$0.001). Two stars above the bar represents significant after Bonferroni correction, one star represents nominal significance (p-value$>$0.05).
