# Supplementary material for: DeCAF: a novel method to identify cell-type specific regulatory variants and their role in cancer risk
Source: Genome Biol. 2022 Jul 8;23:152. doi: 10.1186/s13059-022-02708-9 (PMC9264694; doi:10.1186/s13059-022-02708-9)
Supplement: Supplementary file 3 — Additional file 3 Review history. [file 13059_2022_2708_MOESM3_ESM.docx]

Review History

**First round of review**

**Reviewer 1**

**Were you able to assess all statistics in the manuscript, including the appropriateness of statistical tests used?**

Yes: The statistical tests were described, although clarification would improve the ms.

**Comments to author:**

The authors propose a new method for identifying variants that define eQTL but in a cell type specific manner. These cell fraction eQTLs are identified both by leveraging allelic information among heterozygotes and total expression correlated with genotype (classic eQTL analysis), and finding where the allelic effect fluctuates along with cell fraction based on in silico deconvolution or tumor purity. These are referred to respectively as cell fraction QTLs or tumor specific QTLs. Through simulation it is shown that DeCAF is more powerful than interaction allelic imbalance or interaction QTL alone. DeCAF is then applied to a renal cell carcinoma dataset where it identified more genes than interaction eQTL alone.

Major comments

* How are multiple SNPs aggregated for assessing eQTL / AI concordance? In the Methods it sounds as if a single variant (the most significant) is chosen per gene and cell type. How is information aggregated across variants contributing to the allelic imbalance signal? Does DeCAF assess if the direction of the QTL and the AI is concordant, or does it only combine the p-values? In line 78 it sounded like variants outside the gene were linked to AI signal through haplotype phasing, but then it wasn't clear if QTL effect sizes were ever compared to AI effect sizes. Note that this correlation of eQTL and AI is detailed in the Results and Methods section of Castel et al 2020 (doi:10.1186/s13059-020-02122-z) but not clear here. In line 164 it is mentioned that GTEx eQTL do not use AI signal but actually the haplotype counts are available and can be linked to the GTEx eQTLs, as described in this paper.

* For eQTL analysis, how was expression estimated? What normalization or technical variation correction methods are used? This is relevant when comparing allele specific expression with eQTL effect sizes, if the DeCAF method is attempting to find concordance between these two signals on the level of effect size (this wasn't clear from methods). Methods which have described how to jointly model AI and eQTL effect sizes include TReCASE (doi:10.1111/j.1541-0420.2011.01654.x), RASQUAL (ref 66), and the recent BaseQTL (doi:10.1038/s43588-021-00087-y), and comparison across QTL and allelic fold change has been discussed also by Mohammadi 2017 (doi:10.1101/gr.216747.116). Is DeCAF more comparable to WASP which combines p-values from QTL and AI signal, but DeCAF allows for interaction with cell fraction? Does DeCAF without cell fraction reduce to the combined haplotype test from WASP?

* The cellular fraction is simulated as a uniform (in Methods it says "or from real data" implying in other cases simulated from real data distributions? but which simulation uses which distribution? this also wasn't mentioned as a parameter in line 91 onward in Results). Uniform is an optimistic case for the method, as power depends on the ability to explain variance in the allelic imbalance or QTL effect by variance in cellular fraction. How does the variace of the simulated uniform compare to the variance in the real datasets, and how does power depend on that variance? This should be assessed by simulation but I didn't see that in the manuscript. In S3 it appears that many cell types have small IQR for cell fraction (y-axis), so those cell types would then have low power for detecting cfQTL. This is discussed briefly in Discussion (line 313), but I didn't see any plots directly connecting power to discover cfQTL over the cell fraction variance.

* Cell type heterogeneity can be a technically induced artifact and can be confounded with clinical aspects, e.g. tumor stage, site of sampling, BMI, etc. What limitations or caveats apply given that the cell fraction may correlate with other potential modulators of the genetic effects?

* Does the replication per tissue depend on sample size of the QTL panel? For example, line 182, the strongest replication in GTEx was in blood, is this related to the sample size of the panel defining the blood eQTL?

Minor comments

* Line 130 SNP pairs had r2 < 0.5, squared correlation of what?

* Line 223 "produced the most confident results", what specifically does confident mean here?

* Line 266 "additional power from AI ... due to its count-based nature" - AI signal and QTL signal both are derived from counts of sequenced reads, I don't see how AI is distinguished as specifically count-based. The other point is valid (internal control), as has been discussed by previous AI and QTL combination methods.

* Throughout cfQTL is a cell fraction QTL or eQTL where the effect sizes varies across bulk samples according to cell fraction. It is possible this term could be confused with a locus where genotype is correlated with the cellular composition. E.g. a variant that increases proliferation of a certain cell type in a heterogeneous tissue. Just a consideration.

**Reviewer 2**

**Were you able to assess all statistics in the manuscript, including the appropriateness of statistical tests used?**

Yes: I have the right expertise to evaluate the statistics of the manuscript.

**Comments to author:**

Journal: Genome Biology

Manuscript ID: 221992

Manuscript Title: A novel method to identify cell-type specific regulatory variants and their role in cancer risk

Comments

In the manuscript, entitled, "A novel method to identify cell-type specific regulatory variants and their role in cancer risk", Kalita et al introduced a tool, namely DeCAF, to identify cell type specific QTLs by leveraging total and allelic expression from RNA-seq results. The authors used the tool to identify cell-fraction QTL (cfQTL) and tumor-specific QTLs(tsQTL). In comparison with conventional interaction-expression QTL (ieQTL), more QTL (i.e., cfQTL and tsQTL) were discovered by DeCAF, thereby suggesting the added values of considering allelic expression. Of note, the authors were also able to replicate the identified cfQTL in an independent cohort and proved that the identified cfQTL can prioritize causal variants identified by genome-wide association studies (GWAS). In the end, two examples were also presented to show utility cases of DeCAF. This is a novel work to dissect cell type-specific QTL by leveraging allelic imbalance (AI). The well-written manuscript clearly describes the tool's implementation, evaluation, and usage by providing examples. However, there are several major questions that need to be addressed. In addition, the authors should carefully check the text for spelling and consistent usage of mathematical notations (check more in the minor comments).

Major comments:

I have the following major comments:

1. The application example presented in the manuscript mainly focused on tumor samples. Is the proposed method also applicable to other tissues? It will be beneficial to demonstrate the performance of DeCAF when applying it on other tissues as well, e.g., whole blood or lung (from GTeX)?

2. One of the important steps in eQTL identification is the control of false discovery, which is usually achieved by multiple test corrections. However, the authors used FDR < 0.1 or FDR<0.2 as the thresholds. Authors must explain why such a loose threshold was used instead of a conventional FDR < 0.05. Also, instead of keeping a consistent threshold throughout one manuscript for reporting the main results, why have the authors used different thresholds?

3. AI was the consequence of many mechanisms except ones mentioned in the manuscript, such as nonsense-mediated decay and alternative splicing [DOI: 10.1146/annurev-biodatasci-021621-122219]. However, the authors did not take them into consideration when estimating the AI from bulk RNA-seq. Is this something that the proposed tool should take into account?

4. In the Methods/Allele-specific quantification, the WASP pipeline was used to remove mapping bias. However, the authors did not show if there is still bias in the processed data. A similar figure like the Fig. S9C reported by Zhang et al. [DOI: 10.1126/science.aay3983] is needed to illustrate the removal of bias.

5. Cell type heterogeneity is one important obstacle to dissect cell type specific QTL. To overcome this, the authors considered tumor heterogeneity and somatic alterations (i.e., copy number variation) into account. However, in the Method/Allele-specific quantification, the authors used only germline variants to estimate AI. I'm wondering if this underestimated the AI, since the somatic variants, such as single nucleotide variants and indels, are overrepresented in tumors [DOI: 10.1038/nrg.2015.17]. One possible method to avoid bias is to estimate genotypes from the RNA-seq results. Earlier, this method is reported to perform fairly well. Good reference could be 1) Best Practices Workflows series by GATK, 2) SNP calling from RNA-seq data ... [DOI: 10.1093/nar/gkw655].

6. On page 13, the authors firstly described "cfQTL as the interaction effect between genotype and cell fraction" from a linear regression (line 364). Later they described cfQTL as an AI effect (line 376). Please be clear how exactly "cfQTL" is defined. I assume the first "cfQTL" should be called "eQTL interaction", because in line 386, the authors claimed that "we combined eQTL interaction and cfQTL" as their proposed DeCAF test statistic.

7. On page 13, the authors used "Stouffer's method' to combine the eQTL interaction and cfQTL tests (these tests are independent and so can be combined)".

a) How exactly this was done? Details need to be described in the Method section.

b) Can authors prove that these tests are independent using real data? To me, both tests are based on read counts data from the same individuals, therefore they are not completely independent. In this sense, the assumption of Stoufer's method is invalid.

c) In Figure 2, the authors demonstrated the performance of DeCAF by showing that the DeCAF is superior to "interaction eQTL" and "cfQTL". As the authors described that the proposed DeCAF method actually "combine the eQTL interaction and cfQTL tests", so is the better performance of DeCAF expected already?

I am not sure if this is a fair comparison: a meta-analysis of results from two individual methods is expected to be more powerful than each of them. So, what is the point of showing Fig 2?

8. In the simulation analysis, the performance of the proposed method needs to be comprehensively evaluated for different parameters, e.g., different MAF, different sample sizes, etc. In addition, the performance of the proposed method needs to be evaluated for a proper range of cell proportions. In figure 1, the authors limited their analysis only to cell proportion between 55% to 90%. It is unclear how the method works when the cell proportion is between 10-55%, which commonly happens in the real biological system.

9. In Page 16-17, the authors described how they performed variant enrichment by focusing on "the SNPs with significance in both DeCAF (FDR<20%) and GWAS (p-value<0.001)". Can the authors explain how these thresholds were defined? In GWAS, 0.001 p-value is very loose, which might correspond to many false positives. So how do the enrichment results look like at GWAS's p-value threshold of 10-4, 10-5, 10-6? Do you see a consistent enrichment pattern? If not, does that mean the reported enrichment results are threshold-dependent, thus unreliable? The same question for the DeCAF threshold: Why was such a loose threshold (FDR=20%) used? How do the enrichment results look like when FDR=15%, 10% and 5%? Do you see a consistent enrichment pattern or it is again threshold-dependent, thus unreliable?

Apart from the major comments, I still have a few minor comments.

Minor comments:

- In the pdf file, the figures numbers were not properly displayed. For example, in Line 52: "Figure ??a". Is there an issue with text compilation? It looks like the compiled PDF was not complete (the figure notations were missing), I have to read their bioRxiv version.

- Line 56: AI was introduced again. It was described in line 34 already.

- Line 57 and line 63: mu was used to represent different parameters in these two regression models. This could bring confusion. Please use other different notations.

- Line 128: 3 -> three - Line 136: rs1176895555-A is very rare. I suppose the authors actually meant rs117689555(?)? In addition, I didn't find this gene GOLGABL1, I suppose the authors meant GOLGA6L1?

- The authors should take care of the format of numbers. For instance, line 107, "1753" -> "1,753"

- The authors should maintain consistency while writing. E.g., line 158 "pi1 > 0.59", but the other way in line 239.

- In the "Methods/Allele-specific quantification", the authors used the WASP pipeline to remove mapping bias to reference allele, however, the pipeline requires alignment tool; please clarify which aligner was used.

**Authors Response**

**Point-by-point responses to the reviewers’ comments:**

**Reviewer #1:**

The authors propose a new method for identifying variants that define eQTL but in a cell type specific manner. These cell fraction eQTLs are identified both by leveraging allelic information among heterozygotes and total expression correlated with genotype (classic eQTL analysis), and finding where the allelic effect fluctuates along with cell fraction based on in silico deconvolution or tumor purity. These are referred to respectively as cell fraction QTLs or tumor specific QTLs. Through simulation it is shown that DeCAF is more powerful than interaction allelic imbalance or interaction QTL alone. DeCAF is then applied to a renal cell carcinoma dataset where it identified more genes than interaction eQTL alone.

*We thank the reviewer for their thoughtful feedback and for noting that DeCAF is shown to be more powerful than the state of the art in simulation and application to real data.*

Major comments

* How are multiple SNPs aggregated for assessing eQTL / AI concordance? In the Methods it sounds as if a single variant (the most significant) is chosen per gene and cell type.

*1. Thank you for noting the lack of clarity in how eQTL and AI testing and aggregation is handled. We have clarified how single variant testing is carried out:*

*“Each SNP is tested individually and separately for eQTL and AI. For the eQTL test, reference/alternate allele genotype dosage is used as the independent variable. For the AI test, heterozygous individuals are assigned the aggregated allelic read counts (see above) corresponding to their reference/alternate haplotypes. For both signals only a single distal variant is evaluated in a given test, but RNA-seq reads are aggregated across the gene. Each SNP then has eQTL and AI effects combined via Stouffer’s method (see above) to define the final DeCAF statistic.” [pp.18]*

*We have also clarified how the most significant variant per gene is selected:*

*“Significant cfQTLs in each cell type were determined by identifying the most significant variant for each cell type and gene, applying a gene-level Bonferroni correction. At this point a single SNP per gene and cell-type is then selected (the most significant after Bonferroni correction). This set of SNPs then is further BH (Benjamini-Hochberg False Discovery Rate) corrected across genes, per cell-type.” [pp.19]*

How is information aggregated across variants contributing to the allelic imbalance signal?

*2. Allelic information across multiple variants in a gene is aggregated as in prior work using haplotype phase (van de Geijn et al. 2015; Gusev et al 2019). We have added a brief description to the methods section to go along with the citation: “For each individual, allelic reads from all heterozygous variants within the tested gene are aggregated using haplotype information, under the assumption that the regulatory effect is consistent across the haplotype (van de Geijn et al. 2015; Gusev et al 2019).” [pp.18]*

Does DeCAF assess if the direction of the QTL and the AI is concordant, or does it only combine the p-values?

*3. The DeCAF statistic uses Stouffer’s method, which assesses the direction of the QTL and AI effect in addition to the significance (equivalent to an inverse-variance weighted meta analysis). We have clarified this in the Methods: “Stouffer’s method is the sum of the Z scores (here the test statistics derived from the QTL and AI tests), divided by the square of the number of values input. This combined statistic is equivalent to an inverse-variance weighted meta-analysis between the two associations and thus assumes a shared underlying effect (i.e. effects in opposite directions will become less significant when combined)” [pp.15]*

In line 78 it sounded like variants outside the gene were linked to AI signal through haplotype phasing,

*4. That’s correct, a variant outside a gene is tested for AI signal using the haplotype phase information to the read-carrying variants in the gene. We have clarified this in the Methods: “heterozygous individuals are assigned the aggregated allelic read counts (see above) corresponding to their reference/alternate haplotypes. For both signals only a single distal variant is evaluated in a given test, but RNA-seq reads are aggregated across the gene.” [pp.18]*

but then it wasn't clear if QTL effect sizes were ever compared to AI effect sizes. Note that this correlation of eQTL and AI is detailed in the Results and Methods section of Castel et al 2020 (doi:10.1186/s13059-020-02122-z) but not clear here.

*5. We have clarified that AI and QTL effect sizes are directly compared and meta-analyzed through the Stouffer’s method to compute the DeCAF statistic (see also response 1 above). We also thank the reviewer for highlighting the comparison from Castel et al. 2020 and we have included a scatterplot of the AI/QTL Z-scores for marginal QTLs in the supplement, which shows a similar level of consistency as in prior published work: “The eQTL and AI effects were highly and significantly correlated in the marginal DeCAF results (rho = 0.8, p-value < 2.2^{-16}, Supplement Figure 6).” [pp.5]*

In line 164 it is mentioned that GTEx eQTL do not use AI signal but actually the haplotype counts are available and can be linked to the GTEx eQTLs, as described in this paper.

*6. We have clarified in the text that GTEx has calculated AI separately but these values were not used in their estimation of population-scale eQTL effects.*

* For eQTL analysis, how was expression estimated? What normalization or technical variation correction methods are used?

*7. The hg19 gene expression data was downloaded from firecloud, which is described as being upper-quartile normalized count estimates. This information has been clarified in the methods section. “Total quantified and normalized gene expression (upper-quartile normalized count estimates) was accessed as previously described.” [pp.18]*

This is relevant when comparing allele specific expression with eQTL effect sizes, if the DeCAF method is attempting to find concordance between these two signals on the level of effect size (this wasn't clear from methods).

*8. We have added an evaluation of the concordance between these two signals, demonstrating that they are consistent (see Response 5 above). We note that since the DeCAF statistic uses Stouffer’s method to combine Z-scores (i.e. working on the variance scale) it is not particularly sensitive to the expression normalization.*

Methods which have described how to jointly model AI and eQTL effect sizes include TReCASE (doi:10.1111/j.1541-0420.2011.01654.x), RASQUAL (ref 66), and the recent BaseQTL (doi:10.1038/s43588-021-00087-y), and comparison across QTL and allelic fold change has been discussed also by Mohammadi 2017 (doi:10.1101/gr.216747.116).

*9. We thank the reviewer for these additional references. We have included them in the Introduction.*

Is DeCAF more comparable to WASP which combines p-values from QTL and AI signal, but DeCAF allows for interaction with cell fraction? Does DeCAF without cell fraction reduce to the combined haplotype test from WASP?

*10. Yes, DeCAF does share some similarities to WASP, and without cell fraction they are testing for the same quantity. However, WASP uses a single global overdispersion parameter whereas DeCAF uses local parameters to account for somatic copy number fluctuations (in addition to copy number masking). Additionally, WASP uses a beta-binomial + negative binomial likelihood model for AI/QTL whereas DeCAF uses a beta-binomial regression and standard linear regression followed by Stouffer’s combined method. As noted by the reviewer, we believe the primary innovation of DeCAF is the ability to test for a cell fraction interaction.*

*We have clarified these similarities of the marginal test in the Methods: “For the marginal association without cell fraction interactions and tumor variation adjustment (see above), DeCAF estimates the same underlying quantity as the previously proposed WASP and RASQUAL tests, with minor differences in how the likelihoods are maximized (regression versus grid search).” [pp.19]*

* The cellular fraction is simulated as a uniform (in Methods it says "or from real data" implying in other cases simulated from real data distributions? but which simulation uses which distribution? this also wasn't mentioned as a parameter in line 91 onward in Results).

*11. We apologize for the confusion regarding the simulation parameters. The simulations drawn from a uniform cell fraction were only shown in the Supplement to confirm the basic model assumptions; the main figure results show simulations using real cell fractions sampled from the TIMER inference of TCGA KIRC data. We have clarified in the Results: “Under the alternative hypothesis, DeCAF consistently met or outperformed the power of the conventional interaction eQTL test both under cell fractions generated from a uniform distribution (Supplement Figure 1b) and real cell fractions from TIMER inference in the TCGA KIRC data (Figure 1c) requiring 0.56x fewer samples on average to reach $>$75\% power (Figure 1c).” [pp.4]*

Uniform is an optimistic case for the method, as power depends on the ability to explain variance in the allelic imbalance or QTL effect by variance in cellular fraction. How does the variace of the simulated uniform compare to the variance in the real datasets, and how does power depend on that variance? This should be assessed by simulation but I didn't see that in the manuscript.

*12. We agree that the uniform distribution includes larger cell fractions and less variance than is seen in the real data (which can be seen in the supplement). That is why the main simulation results and figure in the paper uses real cell fractions from the TIMER analysis. We apologize for the confusion on this, and have included the uniform distribution simulation results as a plot in the supplement, and add clarification in the methods and results sections (see also response 11 above).*

In S3 it appears that many cell types have small IQR for cell fraction (y-axis), so those cell types would then have low power for detecting cfQTL. This is discussed briefly in Discussion (line 313), but I didn't see any plots directly connecting power to discover cfQTL over the cell fraction variance.

*13. We have clarified the connection between IQR and discovery power and how well-powered cell types were selected (“To further improve power for testing in the xCell results, we focus on using cell types with a cell fraction score with an interquartile range $>$0.1 and cell types expected to be found in kidney tissue (Supplement Figure 4)” [pp.17]) and have also emphasized the simulation results using real cell fraction distributions (Figure 1c; see also Response 12 above).*

* Cell type heterogeneity can be a technically induced artifact and can be confounded with clinical aspects, e.g. tumor stage, site of sampling, BMI, etc. What limitations or caveats apply given that the cell fraction may correlate with other potential modulators of the genetic effects?

*14. We thank the reviewer for this comment. Any other clinical factor that's correlated with the cell fraction could be the causal mediator. However, this will only impact the mechanistic interpretation of the association, the QTL-cell-fraction interaction will remain statistically valid . This caveat has been added to the Discussion. “Fifth, any other clinical factor that's correlated with the cell fraction could be the causal mediator (for example - tumor stage, site of sampling and BMI), which could be further investigated by incorporating additional relevant interaction terms.” [pp.13]*

* Does the replication per tissue depend on sample size of the QTL panel? For example, line 182, the strongest replication in GTEx was in blood, is this related to the sample size of the panel defining the blood eQTL?

*15. Thank you for raising this important point. Replication rate will depend on statistical power in the target dataset, which is a function of sample size and data quality. Though sample sizes were relatively high for all tissues evaluated (N>300) data/tissue quality may differ substantially, so interpretation about tissue matching (or lack thereof) should be made with caution. We have added these caveats to the Results section. [pp.7]*

Minor comments

* Line 130 SNP pairs had r2 < 0.5, squared correlation of what?

*We have clarified in the text that this is referring to LD.*

* Line 223 "produced the most confident results", what specifically does confident mean here?

*We clarify that this is referring to the smallest 95% confidence intervals.*

* Line 266 "additional power from AI ... due to its count-based nature" - AI signal and QTL signal both are derived from counts of sequenced reads, I don't see how AI is distinguished as specifically count-based. The other point is valid (internal control), as has been discussed by previous AI and QTL combination methods.

*We regret the confusion, the intended point was only that the “internal control” nature of AI means statistical power can scale with the number of reads rather than just the number of individuals. We have clarified this statement in the Discussion.*

* Throughout cfQTL is a cell fraction QTL or eQTL where the effect sizes varies across bulk samples according to cell fraction. It is possible this term could be confused with a locus where genotype is correlated with the cellular composition. E.g. a variant that increases proliferation of a certain cell type in a heterogeneous tissue. Just a consideration.

*Thank you for spotting, we have clarified this when introducing the term.*

**Reviewer #2:**

Comments

In the manuscript, entitled, "A novel method to identify cell-type specific regulatory variants and their role in cancer risk", Kalita et al introduced a tool, namely DeCAF, to identify cell type specific QTLs by leveraging total and allelic expression from RNA-seq results. The authors used the tool to identify cell-fraction QTL (cfQTL) and tumor-specific QTLs(tsQTL). In comparison with conventional interaction-expression QTL (ieQTL), more QTL (i.e., cfQTL and tsQTL) were discovered by DeCAF, thereby suggesting the added values of considering allelic expression. Of note, the authors were also able to replicate the identified cfQTL in an independent cohort and proved that the identified cfQTL can prioritize causal variants identified by genome-wide association studies (GWAS). In the end, two examples were also presented to show utility cases of DeCAF. This is a novel work to dissect cell type-specific QTL by leveraging allelic imbalance (AI). The well-written manuscript clearly describes the tool's implementation, evaluation, and usage by providing examples. However, there are several major questions that need to be addressed. In addition, the authors should carefully check the text for spelling and consistent usage of mathematical notations (check more in the minor comments).

*We thank the reviewer for their feedback and for noting the novelty of the methods and well written manuscript.*

Major comments:

I have the following major comments:

1. The application example presented in the manuscript mainly focused on tumor samples. Is the proposed method also applicable to other tissues? It will be beneficial to demonstrate the performance of DeCAF when applying it on other tissues as well, e.g., whole blood or lung (from GTeX)?

*16. Thank you for this suggestion. DeCAF is easily applied to other types of datasets, with cancer used here as a particularly challenging example (due to tumor impurity and structural variation). As a demonstration, we have applied DeCAF to (n=70) matched normal samples from the KIRC dataset and showed overall improved power for DeCAF versus the conventional ieQTL. This is described in the Results section. “DeCAF can also identify cell-fraction effects in normal tissue. As a demonstration, we applied DeCAF to matched kidney normal RNA-seq data from the same TCGA KIRC cohort (N=70). This much smaller cohort still yielded a total of 287 significant cfQTL eGenes identified by DeCAF, compared to 201 cfQTL eGenes by conventional ieQTL (FDR 10%). A larger proportion of the identified effects were shared (71% shared cfQTLs for normal versus 32% for tumor; Supplement Figure 7), likely due to the much lower sample size and lower number of heterozygous individuals for the AI component as can be seen in simulations (Supplement Figure 1b).“ [pp.7]*

*We also considered applying DeCAF to the GTEx study, which had previously estimated allele-specific signals. However, the public allelic imbalance data has been de-identified to remove any linkage to the specific germline alleles (i.e. only arbitrary haplotype1 / haplotype2 results are provided for each sample), and analysis with DeCAF would require accessing the (restricted) underlying genotype data and re-integrating the allelic counts, which is out of scope for this analysis. We have noted this analysis as potential future work in the Discussion.*

2. One of the important steps in eQTL identification is the control of false discovery, which is usually achieved by multiple test corrections. However, the authors used FDR < 0.1 or FDR<0.2 as the thresholds. Authors must explain why such a loose threshold was used instead of a conventional FDR < 0.05. Also, instead of keeping a consistent threshold throughout one manuscript for reporting the main results, why have the authors used different thresholds?

*17. Thank you for raising this point. 10% FDR is not uncommonly used to report eQTL results where the effect sizes are expected to be weak (for example, Kim-Hellmuth et al. 2020 Science used a 40% FDR cutoff for reporting ieQTLs; and GTEx et al. 2017 Nature used a 10% FDR cutoff for reporting trans-QTLs). We have conducted additional analyses at more stringent FDR cutoffs and did not find the results changed substantially. This has been added as Supplementary Figure 8.*

*The only other instance where a looser FDR threshold was used is in the integration of DeCAF results with GWAS SNPs, due to the kidney cancer GWAS study being very small and having few genome-wide significant loci intersecting with any QTLs. However, we provide results from all thresholds in Supplementary Figure 11a and note the use of a less strict threshold for this secondary analysis in the Results.*

3. AI was the consequence of many mechanisms except ones mentioned in the manuscript, such as nonsense-mediated decay and alternative splicing [DOI: 10.1146/annurev-biodatasci-021621-122219]. However, the authors did not take them into consideration when estimating the AI from bulk RNA-seq. Is this something that the proposed tool should take into account?

*18. We thank the reviewer for mentioning this review paper. We agree that AI can be the consequence of many mechanisms such as nonsense-mediated decay, imprinting, post-translational effects and alternative splicing. By assuming consistent AI/QTL effects in DeCAF, our approach may lose power when these deviating effects are present. However, prior work has found that, on average, AI and QTL effect sizes are highly concordant and are thus likely capturing the same genetic mechanisms in the population (Castel et al. 2020). We have also now confirmed this consistency of effect sizes in our data: “The eQTL and AI effects were highly and significantly correlated in the marginal DeCAF results ($\rho$ = 0.8, $p$-value $<$ $2.2^{-16}$, Supplement Figure 6).” (see also Response 5 above).*

*Our real data analyses also show that the combined DeCAF model achieves the largest power (with comparable external replication rate), further supporting these assumptions. We have added this caveat and citation to the Discussion [pp.13].*

4. In the Methods/Allele-specific quantification, the WASP pipeline was used to remove mapping bias. However, the authors did not show if there is still bias in the processed data. A similar figure like the Fig. S9C reported by Zhang et al. [DOI: 10.1126/science.aay3983] is needed to illustrate the removal of bias.

*19. Thank you for the suggestion. We have included a plot similar to Zhang et al to show no apparent mapping bias. We have a much larger study than Zhang et al. so it’s harder to see the shape of the distribution, which is why we supplemented it with violin plots. This is now included as Supplementary Figure 5.*

5. Cell type heterogeneity is one important obstacle to dissect cell type specific QTL. To overcome this, the authors considered tumor heterogeneity and somatic alterations (i.e., copy number variation) into account. However, in the Method/Allele-specific quantification, the authors used only germline variants to estimate AI. I'm wondering if this underestimated the AI, since the somatic variants, such as single nucleotide variants and indels, are overrepresented in tumors [DOI: 10.1038/nrg.2015.17]. One possible method to avoid bias is to estimate genotypes from the RNA-seq results. Earlier, this method is reported to perform fairly well. Good reference could be 1) Best Practices Workflows series by GATK, 2) SNP calling from RNA-seq data ... [DOI: 10.1093/nar/gkw655].

*20. Thank you for noting a potential bias introduced by somatic single nucleotide variants (SNVs). In principle, somatic variants will introduce additional noise into the analysis by sporadically knocking out or activating driver genes, but they would not be expected to lead to false positives in the absence of germline-somatic interactions. Fortunately, somatic SNV calling has already been performed on this dataset by TCGA using whole-exome sequencing of the tumor and normal, allowing us to investigate this question directly. We have added this analysis to the Results: “In principle, somatic variants will introduce additional noise into the analysis by sporadically knocking out or activating driver genes, but they would not be expected to lead to false positives in the absence of germline-somatic interactions. Analyzing somatic SNV calling data from TCGA whole-exome sequencing, we found that a total of 96 genes had >10 somatic mutation carriers (sufficient to influence population-scale QTL discovery), which overlapped only 9/3,654 of the significant marginal DeCAF eGenes we previously identified. For these 9 genes that were significant marginal DeCAF eGenes and had >10 SNV carriers, we tested each lead SNP for a germline-somatic interaction by adding somatic carrier status as a binary interaction term (similar to how purity is included as an interaction term for tsQTL analysis). None of the 9 interaction terms were statistically significant after Bonferroni correction, indicating no significant differences in AI/QTL effect size between carriers and non-carriers. In sum, we find that recurrent SNVs are unlikely to influence the overall DeCAF results due to the generally low number of highly recurrently mutated eGenes, and a formal interaction test of these genes did not modify the associations. “ [pp.6]*

6. On page 13, the authors firstly described "cfQTL as the interaction effect between genotype and cell fraction" from a linear regression (line 364). Later they described cfQTL as an AI effect (line 376). Please be clear how exactly "cfQTL" is defined. I assume the first "cfQTL" should be called "eQTL interaction", because in line 386, the authors claimed that "we combined eQTL interaction and cfQTL" as their proposed DeCAF test statistic.

*21. We apologize for the lack of clarity in these terms. We have fixed this in the Methods section where they are described as “cell fraction ieQTL effect” and “cell fraction iAI effect” and then “we combined the cell fraction ieQTL and iAI tests”.*

7. On page 13, the authors used "Stouffer's method' to combine the eQTL interaction and cfQTL tests (these tests are independent and so can be combined)".

a) How exactly this was done? Details need to be described in the Method section.

*22. Stouffer’s method is the sum of the z scores divided by the square of the number of values input (equivalent to an inverse-variance weighted meta-analysis between the two associations). We have included this in the Methods section.*

b) Can authors prove that these tests are independent using real data? To me, both tests are based on read counts data from the same individuals, therefore they are not completely independent. In this sense, the assumption of Stoufer's method is invalid.

*23. Thank you for noting this. Our assumption that the total and allelic signals are independent is the same as that made by prior approaches for combining QTL/AI data, including including TReCASE (Sun et al 2012), RASQUAL (Kumasaka et al 2016) and BaseQTL (Vigorito et al 2021). Even though expression is used from the same individuals, the tests are independent because the tested independent variable is different: eQTLs use the variance between the 0/1/2 genotypes, whereas AI uses the variance within the 1 genotype (between alleles). We note that this is further supported by our simulations, which simulate consistent allelic effects in the same individuals and show that the test is well controlled under the null (Supp Figure 1 and 2). We have added clarifications and additional references regarding this assumption to the Methods. [pp.15]*

c) In Figure 2, the authors demonstrated the performance of DeCAF by showing that the DeCAF is superior to "interaction eQTL" and "cfQTL". As the authors described that the proposed DeCAF method actually "combine the eQTL interaction and cfQTL tests", so is the better performance of DeCAF expected already?

I am not sure if this is a fair comparison: a meta-analysis of results from two individual methods is expected to be more powerful than each of them. So, what is the point of showing Fig 2?

*24. We agree that DeCAF is expected to increase power if model assumptions hold, since it is incorporating an additional source of genetic signal. However, these assumptions are not a priori guaranteed; if AI and QTL effects are not shared or AI effects are unexpectedly noisy then DeCAF would identify fewer significant effects than interaction eQTL (by adding noise to signal). The purpose of Figure 2 is to demonstrate that these assumptions indeed hold and lead to higher power in real data. We have clarified this when referring to Figure 2 [pp.5].*

8. In the simulation analysis, the performance of the proposed method needs to be comprehensively evaluated for different parameters, e.g., different MAF, different sample sizes, etc. In addition, the performance of the proposed method needs to be evaluated for a proper range of cell proportions. In figure 1, the authors limited their analysis only to cell proportion between 55% to 90%. It is unclear how the method works when the cell proportion is between 10-55%, which commonly happens in the real biological system.

*25. We regret the confusion about the simulation analysis, which had already addressed most of these concerns but presented the findings very tersely in the Results. Taking each point in turn:*

*“different MAF”*

*We simulated MAFs ranging from 0.1-0.5. (Supplement Figure 1 and 2)*

*“different sample sizes”*

*We simulated sample sizes ranging from 0 to 1000 (Figure 1c, Supplement Figure 1 and 2)*

*“etc”*

*We additionally simulated a range of variant effect sizes (allelic fraction, see below) and read depth (Figure 1c, Supplement Figure 1 and 2; read depth unshown).*

*“In addition, the performance of the proposed method needs to be evaluated for a proper range of cell proportions”*

*We simulated using real cell fractions from the TIMER deconvoluted data to evaluate the most realistic range of cell proportions possible (Figure 1c).*

In figure 1, the authors limited their analysis only to cell proportion between 55% to 90%. It is unclear how the method works when the cell proportion is between 10-55%, which commonly happens in the real biological system.

*26. We regret the confusion, Figure 1 shows results from simulations using real cell fractions from the TIMER deconvoluted data. We believe the reviewer is misinterpreting the 55-90% panels (rows of Figure 1c) to be cell fraction, these actually represent the allelic fraction i.e. the causal variant effect size. The cell fractions are shown in the columns (i.e. B_cell, CD4_Tcell, etc) and are taken from the corresponding real TIMER cell fractions. Simulations with cell fractions sampled from the uniform distribution (which was not restricted to 55-90%) are only presented in Supplementary Figure 1, primarily to show face-validity of the simulation framework.*

*We regret the confusion, and have expanded the simulation Results section and Figure 1 legend with additional clarifications. “In particular, we sampled cell fractions from the real TIMER-inferred fractions in TCGA across 6 cell-types, to investigate the performance of this approach under realistic cell proportions.” [pp.4]*

9. In Page 16-17, the authors described how they performed variant enrichment by focusing on "the SNPs with significance in both DeCAF (FDR<20%) and GWAS (p-value<0.001)". Can the authors explain how these thresholds were defined? In GWAS, 0.001 p-value is very loose, which might correspond to many false positives. So how do the enrichment results look like at GWAS's p-value threshold of 10-4, 10-5, 10-6? Do you see a consistent enrichment pattern? If not, does that mean the reported enrichment results are threshold-dependent, thus unreliable? The same question for the DeCAF threshold: Why was such a loose threshold (FDR=20%) used? How do the enrichment results look like when FDR=15%, 10% and 5%? Do you see a consistent enrichment pattern or it is again threshold-dependent, thus unreliable?

*27. We agree that a loose GWAS significance threshold will introduce variants with more noise (at the benefit of being able to test polygenic, sub-threshold variants for enrichment) and resorted to a looser threshold because the RCC GWAS was relatively small and underpowered for testing of genome-wide significant variants. We report the enrichment results across a grid of GWAS p-value cutoffs and FDR thresholds in Figure S11. We note that the key enrichments were consistent across these thresholds: enrichment for (TIMER) dendritic cells was nominally significant for 9/9 (with enough variants to perform the test) 9/9 (with enough variants to perform the test) threshold choices and enrichment for all (TIMER) cfQTLs was nominally significant for 9/9 (with enough variants to perform the test) threshold choices (these are the two key enrichments we highlight in the Results). As such, our main findings are largely robust to choice of significance threshold. We also note that our choice of thresholds is much more stringent than that used by Kim-Hellmuth et al 2020 Science for a similar enrichment analysis (40% FDR for cfQTLs; p ≤ 0.05 for GWAS SNPs). We have added these additional caveats to the results [pp.9].*

Apart from the major comments, I still have a few minor comments.

Minor comments:

- In the pdf file, the figures numbers were not properly displayed. For example, in Line 52: "Figure ??a". Is there an issue with text compilation? It looks like the compiled PDF was not complete (the figure notations were missing), I have to read their bioRxiv version.

*We apologize for the formatting issues. We have double checked the new compilation before resubmitting.*

- Line 56: AI was introduced again. It was described in line 34 already.

*We have removed the double reference.*

- Line 57 and line 63: mu was used to represent different parameters in these two regression models. This could bring confusion. Please use other different notations.

*We have changed the notation of mu for the AI test to now be mu_a, so as to avoid confusion.*

- Line 128: 3 -> three - Line 136: rs1176895555-A is very rare. I suppose the authors actually meant rs117689555(?)? In addition, I didn't find this gene GOLGABL1, I suppose the authors meant GOLGA6L1?

*We apologize for the typo in the gene name and rsID in the text. It is GOLGA6L1 and rs117689555.*

- The authors should take care of the format of numbers. For instance, line 107, "1753" -> "1,753"

*We have fixed the number format.*

- The authors should maintain consistency while writing. E.g., line 158 "pi1 > 0.59", but the other way in line 239.

*We have fixed the pi1 notation consistency.*

- In the "Methods/Allele-specific quantification", the authors used the WASP pipeline to remove mapping bias to reference allele, however, the pipeline requires alignment tool; please clarify which aligner was used.

*We have added alignment tool information to the methods section.*

**Second round of review**

**Reviewer 1**

The authors have addressed my comments/concerns in my previous review.
